# The Challenges of AI Applications in Health Care: Qualitative Study

**DOI:** 10.2196/92979

**Published:** 2026-08-18

**Authors:** Lilin Qiu, Shuyao Li, Yumei Liang, Shuyu Lu, Pinyue Tao

**Affiliations:** 1 Department of Nursing The Second Affiliated Hospital of Guangxi Medical University Nanning, Guangxi China

**Keywords:** AI, application challenges, artificial intelligence, health care sector, qualitative research, thematic analysis

## Abstract

**Background:**

The application of AI in clinical settings is becoming increasingly widespread, exerting a profound impact on health care professionals’ work patterns, decision-making efficiency, and clinical practice. However, there is currently a lack of systematic research on health care professionals’ actual experiences, barriers to use, and attitudes toward the clinical application of AI. AI has shown great potential to streamline clinical workflows and improve service quality, yet its real-world adoption faces notable obstacles.

**Objective:**

This study aimed to comprehensively explore the multifaceted challenges encountered during the implementation of AI in routine health care work. The findings are intended to offer empirical evidence and practical references for improving the deployment, management, and popularization of AI tools in clinical settings.

**Methods:**

We adopted a qualitative design with inductive thematic analysis. Through purposive sampling, 23 health care workers with diverse job roles, professional titles, and work experience were recruited for one-on-one semistructured interviews. Two researchers independently performed data coding and cross-checked the results to ensure intercoder reliability. NVivo (version 15.0; Lumivero) software was used to organize the qualitative data and support thematic analysis.

**Results:**

All data were fully transcribed. The interviews identified 5 themes and 13 subthemes related to the challenges of applying AI in the health care sector. The five themes are as follows: (1) construction of AI trust mechanisms and shaping of rational cognition, (2) AI-assisted decision-making models and health care collaboration, (3) AI user experience, (4) barriers to AI use and risk concerns, and (5) attitudes toward AI substitution and perception of core health care value. Participants generally recognized the value of AI in clinical work, yet they highlighted multiple practical barriers and potential risks.

**Conclusions:**

The application of AI in health care is shaped by the interplay of complex factors spanning technology, organization, cognition, and regulation. A balanced perspective is essential to ensure that AI integration enhances rather than undermines clinical practice. While AI has great potential to boost efficiency and support clinical decision-making, it also highlights risks such as overreliance, cognitive decline, and the long-term deterioration of professional skills and independent clinical reasoning. Accordingly, efforts should be made to optimize the design and deployment of AI tools, along with a fundamental rethinking of professional training and governance frameworks. Future work should prioritize developing intelligence-augmenting rather than intelligence-replacing systems. Critical evaluation of AI outputs needs to be incorporated into clinical education, and relevant policies should be formulated to prevent cognitive complacency. The ultimate goal is to achieve a sound, sustainable, and people-centered integration of AI into routine clinical practice.

## Introduction

AI technology is advancing rapidly and is being increasingly applied in various scenarios within the health care sector, including areas such as image-assisted diagnosis, intelligent electronic medical record entry, nursing plan formulation, and intelligent decision support [1,2]. The introduction of AI tools in the health care sector is expected to enhance the efficiency of medical services, optimize diagnostic and treatment processes, and alleviate the workload of health care professionals, making them a key driver of high-quality development in the health care industry. However, based on current results, the application of AI in health care has not fully met expectations [3]. Some health care professionals have limited acceptance of and engagement with AI tools, and there is a tendency for AI products to be easy to introduce but difficult to implement effectively. Existing research in this area has largely focused on the technical optimization of AI tools, and evaluations of their effectiveness tend to be limited and fragmented. There is a lack of systematic and in-depth analysis of the challenges associated with applying AI technology in the health care sector [4], and there is an even greater shortage of research based on a qualitative perspective [5]. Compared to quantitative surveys, qualitative research is better able to accurately capture researchers’ genuine thoughts, inner concerns, and value judgments regarding the application of AI technology, and to deeply analyze the psychological mechanisms and sociocultural factors underlying these challenges [6]. This is precisely where the current research gap lies. Existing qualitative studies have not yet fully explored the challenges, practical needs, genuine experiences, and psychological processes of health care professionals in applying AI [7], making it difficult to comprehensively reveal the multifaceted causes and underlying logic of the challenges associated with AI applications in the health care sector.

As national policies continue to promote the AI + health care strategy, the integration of AI technology with the health care industry has deepened further. However, the challenges associated with its application have become increasingly prominent [8]. Failing to promptly identify the manifestations, underlying causes, and influencing factors of these challenges will severely impede the sustainable and effective development of AI in health care, thus hindering the achievement of national health and wellness strategic goals. Against this backdrop, it is imperative to systematically investigate the practical challenges of large-scale AI application in the health care sector and thoroughly examine the underlying factors, including stakeholders’ perceptions, technical limitations, institutional gaps, and social factors.

Therefore, this study used an inductive thematic analysis approach to conduct semistructured, in-depth interviews with health care professionals. The aim was to fill gaps in existing research, refine the research framework for AI technology applications in health care, and provide empirical support for relevant authorities in formulating supportive policies, regulatory standards, and ethical guidelines for AI in health care. Furthermore, this study sought to offer targeted recommendations for the optimization and upgrading of AI health care products and the enhancement of health care professionals’ application capabilities. Thereby, it promotes the standardized and sustainable development of AI in health care and contributes to the high-quality development of the health care industry and the construction of a Healthy China.

## Methods

### Research Design

This study used a qualitative research design based on inductive thematic analysis [9,10] and was reported in full compliance with COREQ (Consolidated Criteria for Reporting Qualitative Research) [11]. Thematic analysis is a data-driven qualitative analysis method that identifies themes directly from raw data. Coding and themes emerge naturally from participants’ narratives, ensuring that the research findings are grounded in authentic experiences. The study used semistructured interviews as the data collection method, focusing on 4 dimensions: current AI usage, perceived challenges, optimization needs, and future prospects, to ensure the comprehensiveness and depth of the data.

### Research Participants

Through purposive sampling, a total of 23 health care professionals were recruited, varying in occupation, professional title, and years of experience. This study primarily used WeCom [12] for online recruitment. The corresponding author selected participants using purposive sampling. A total of 25 potential participants were contacted; 2 declined due to time constraints, resulting in a final sample of 23 participants. To ensure sample diversity, we intentionally recruited participants with varied professional backgrounds, years of work experience, and levels of AI usage experience. The recruitment process was as follows: first, the corresponding author screened eligible participants according to the predefined inclusion and exclusion criteria. Recruitment was conducted in the departments of general medicine, nursing, and medical technology. Potential participants were then contacted via WeCom, and the study purpose and procedures were explained in detail. Participants who agreed to take part signed a written informed consent form. Interview dates and locations were scheduled at mutually convenient times. Eligibility was reconfirmed before each interview. To minimize selection bias, we purposively sampled participants with varied professional backgrounds, work experience, and AI exposure levels. Recruitment continued until data saturation was reached, meaning no new codes or themes emerged in consecutive interviews [13]. No members of the research team had prior personal contact or collaborative relationships with any enrolled participants before the recruitment and interview stage. Participants were screened solely via the hospital’s internal WeCom directory without any preexisting acquaintance.

Inclusion criteria were as follows: (1) working in relevant departments of medical institutions with experience using AI tools (AI use refers to the direct application of clinical AI tools in daily work, including reviewing AI-generated outputs, making AI-assisted decisions, or operating AI systems), (2) able to clearly articulate personal usage experiences and perspectives, and (3) voluntarily participating in this interview and signing an informed consent form.

Exclusion criteria were as follows: (1) lack of AI tool usage experience (rendering participants unable to provide meaningful interview information), and (2) inability to complete the full interview due to reasons such as a heavy workload or physical discomfort (to ensure that participants were in good physical condition and maintained focus throughout the interview, enabling them to express their views fully and honestly, thereby ensuring the quality of the interview data).

### Data Collection

The interviews were conducted between September and December 2025 as in-person, face-to-face sessions in quiet settings such as hospital conference rooms and university faculty offices. They were led by a professional researcher who had received systematic training in qualitative research. The interview guide was developed based on the existing literature and the study objectives, focusing on practical experiences, perceived challenges, attitudes, and implementation recommendations related to clinical AI (details are provided in Multimedia Appendix 1). All interviews were anonymously audio-recorded with prior consent. Each interview lasted approximately 20 to 30 minutes. Field notes were taken immediately after each interview to capture nonverbal cues and contextual details. Within 24 hours of each interview, 2 research team members transcribed the recordings into written transcripts and cross-checked them. Selected participants received partial transcripts and preliminary analytical outcomes to conduct member checking for result validation. A total of 23 valid interview transcripts were collected, amounting to approximately 86,000 words.

### Data Analysis

To enhance transferability, we provided detailed descriptions of participants, clinical settings, and data collection procedures, enabling readers to assess the applicability of findings to similar clinical contexts. Data analysis was conducted following the principles of reflexive thematic analysis by Braun and Clarke [9]; updated guidance from Braun and Clarke [10] was also adopted, prioritizing interpretive credibility and analytic confirmability rather than positivist reliability measures. This approach aligns with interpretive qualitative values, emphasizing context-dependent meaning and treating researcher subjectivity as an interpretive resource rather than a limitation. Data organization and coding were conducted using NVivo software (version 15.0; Lumivero) [14]. A comprehensive analytic audit trail was maintained throughout the analysis, with coding decisions, theme development processes, and iterative reflections documented in a reflexive journal. Two researchers independently imported interview transcripts into NVivo 15 for organization and management. After repeated familiarization with the raw data, the 2 researchers independently performed reflexive coding and thematic development on all transcribed interviews and reached consensus through iterative peer discussion, without calculating quantitative agreement. Initial open codes were extracted verbatim from the interview content, and the codebook was continuously updated and finalized through regular research team discussions. Data collection and analysis proceeded sequentially until data saturation was achieved, defined as the point at which no new themes or subthemes emerged.

### Ethical Considerations

Ethical approval was obtained from the Ethics Committee of the Second Affiliated Hospital of Guangxi Medical University under approval number 2025-KYC(0531). The participants provided their written informed consent to participate in this study. Data were collected anonymously throughout the study. As compensation for participating in the study, each participant was given a blue-black ballpoint pen, the type commonly used for written records. This research project was funded by the Key Projects in Philosophy and Social Sciences at Guangxi Medical University.

## Results

### Demographic Characteristics of the Participants

The 23 participants in this study represented a range of positions, professional titles, and years of service. Among them, 9 were male, and 14 were female, with the majority aged between 31 and 40 years. Their demographic characteristics are provided in Table 1.

**Figure 1:**
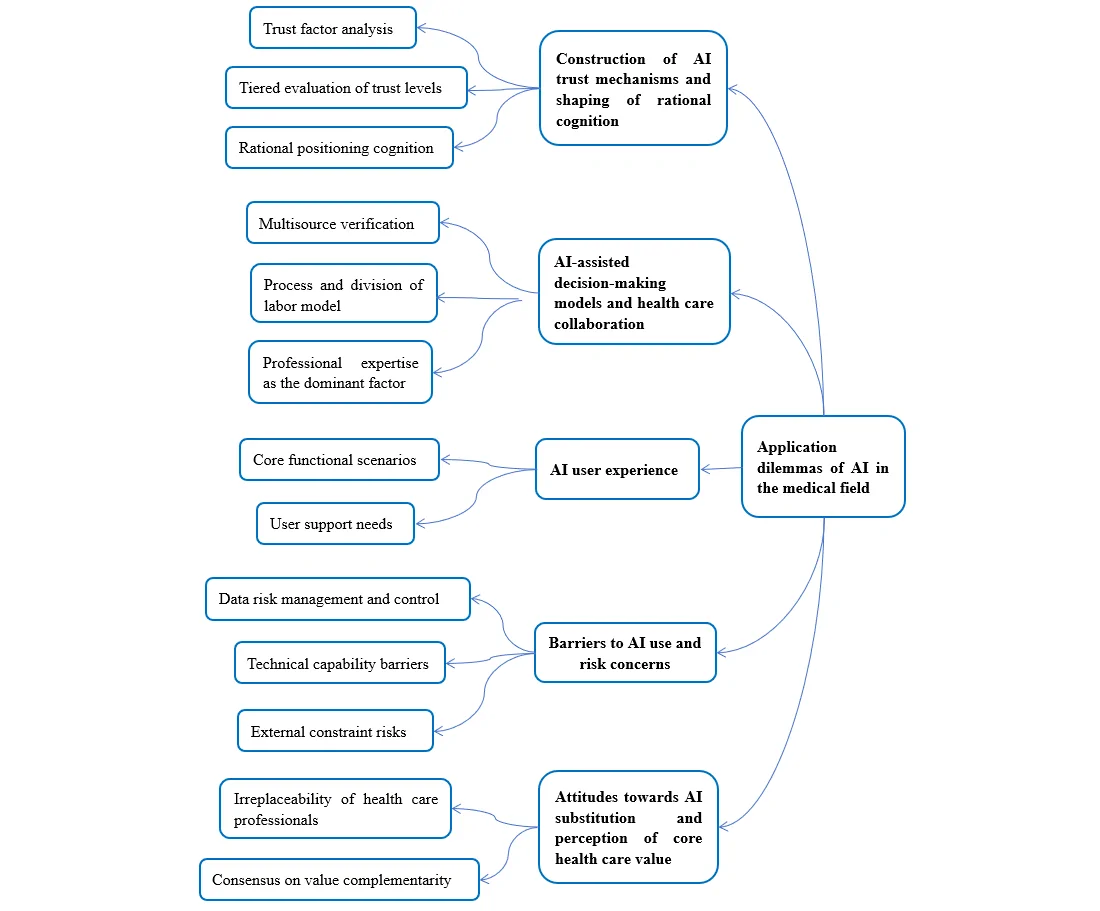
Thematic structure of clinicians’ perceptions of AI use in clinical work (5 main themes and 13 corresponding subthemes).

Through open coding of 23 interview transcripts, 323 raw statements were extracted. After conceptual refinement, 89 initial concepts were identified. These initial concepts were categorized and organized, with duplicates and ambiguous concepts removed. The data ultimately yielded 5 themes, each encompassing several subthemes, totaling 13 subthemes, as illustrated in Figure 1.

**Table 1:** Demographic characteristics of respondents.

Characteristic		Frequency, n (%)
Age group (years)		
	≤30	6 (26.1)
	31-40	12 (52.2)
	41-50	3 (13.0)
	≥51	2 (8.7)
Sex		
	Male	9 (39.1)
	Female	14 (60.9)
Highest education level		
	Bachelor’s degree	11 (47.9)
	Master’s degree	9 (39.1)
	Doctorate degree	3 (13.0)
Job category		
	Physician	9 (39.1)
	Nurse	10 (43.5)
	Medical technician	2 (8.7)
	Other (university faculty)	2 (8.7)
Professional title level		
	Junior	3 (13.0)
	Intermediate	11 (47.9)
	Associate senior	4 (17.4)
	Senior	5 (21.7)
Work experience (years)		
	≤5	7 (30.4)
	6-10	5 (21.8)
	11-20	4 (17.4)
	≥21	7 (30.4)
Common AI tool types^a^		
	Nursing planning	11 (47.9)
	Imaging-assisted diagnosis	8 (34.8)
	Electronic medical record intelligent entry	9 (39.1)
	Other (research, scheduling, and decision support)	10 (43.5)
Usage frequency		
	Multiple times daily	6 (26.1)
	Several times per week	11 (47.8)
	Several times per month	6 (26.1)
First exposure to AI		
	Before 2020	4 (17.4)
	2020-2022	7 (30.4)
	2023-2024	12 (52.2)

^a^Percentages may sum to more than 100% because respondents could select multiple AI tool types.

### Theme 1: Constructing AI Trust Mechanisms and Shaping Rational Perceptions

#### Overview

Centered on the core theme of “building AI trust mechanisms and shaping rational perceptions,” this study identifies 3 major subdimensions. The findings reveal that health care professionals’ trust in AI is not a simple binary judgment based on a single dimension. Instead, it is a dynamic and multifaceted cognitive system formed through AI’s technical capabilities, application scenarios, and their own professional backgrounds. Furthermore, health care professionals exhibit a cautious and balanced rational cognitive profile. This cognitive framework not only lays the foundation for trust mechanisms but also sets limits on the extent of trust, thereby providing empirical evidence for the systematic construction of AI trust mechanisms.

#### Subtheme 1: Analysis of Trust Factors

Health care professionals’ trust in AI is built on its technical performance. However, it is also influenced by the characteristics of different application scenarios, and these 2 factors jointly shape the development of trust. Among these, the accuracy and reliability of AI are the core prerequisites for trust. The research data indicate that the accuracy of AI output is a critical factor in building trust among health care professionals; any deviation in results or confusion in information leads to a sharp decline in trust. Furthermore, in the specialized field of health care, the requirements for accuracy are even more stringent. This suggests that accuracy is the condition for AI to cross the threshold of trust, and technical performance lacking in accuracy will become a stumbling block to establishing a trust mechanism.

When I use AI to search for literature and find citation sources, the results are all over the place and inaccurate, which undermines my trust in it.

A3

The key factor determining trust is the suitability of the application scenario. The level of trust in the same AI technology varies across different application scenarios, and medical professionals adjust their trust standards based on the risk level of the scenario. In low-risk scenarios, there is typically a higher tolerance for AI inaccuracies, but in high-risk scenarios involving professional diagnoses, it is difficult to establish trust in AI. Thus, the risk attributes of a scenario determine the criteria for measuring trust, and scenario adaptability becomes a critical boundary condition for the implementation of trust mechanisms. In addition, our results indicated that employees with longer tenure and higher professional titles exhibited lower levels of trust.

When it comes to handling simple tasks, I trust it completely; but when dealing with complex matters, especially academic issues, AI may suffer from ‘AI hallucination,’ so I have less confidence in it.

A8

#### Subtheme 2: Tiered Evaluation of Trust Levels

Health care professionals exhibit a moderate level of trust in AI. Their evaluation process relies on multisource verification and dynamic adjustment. No extreme evaluations, such as complete trust or complete distrust, emerged during the interviews; this nuanced pattern suggests that health care professionals’ trust in AI is performance-based and subject to dynamic adjustment.

My level of trust is moderate. It depends on the question I’m asking; I’ll cross-reference the information with what I find on other websites and make a comprehensive judgment before deciding whether to trust it.

A7

Medical professionals do not rely solely on AI-generated results. Instead, they build a trust evaluation system through multisource comparisons and professional verification. This reflects a rational approach to AI and establishes a self-trust evaluation mechanism based on comprehensive verification of information from multiple sources.

Go to China National Knowledge Infrastructure (CNKI) or other platforms to search for the latest literature, compare the AI results with the content of the literature, and see if there are any discrepancies.

A6

For complex medical diagnoses, we must take into account individual patient differences. This relies on the clinical experience we’ve accumulated over many years.

A13

#### Subtheme 3: Rational Positioning Cognition

Health care professionals exhibit a rational attitude toward AI. They neither blindly accept nor outright reject it; instead, they adopt a cautious and appropriate approach to adaptation. This cognitive stance functions as a trust mechanism, ensuring the safety and reliability of AI applications and guiding their proper use. A balanced perspective is attained through this dual attitude of neither blindly adoring nor completely dismissing AI.

I believe we should maintain a critical view of AI. Different AI systems have distinct characteristics, so we need to select the appropriate one according to our specific requirements.

A10

### Theme 2: AI-Assisted Decision-Making Models and Health Care Collaboration

#### Overview

Our results indicated that the guiding principles for AI application are as follows: first, professional oversight; second, cautious use. Health care professionals rely on their own expertise and practical experience to provide professional oversight of AI applications, establishing a consistent pattern of AI-assisted, human-driven decision-making.

#### Subtheme 1: Multisource Verification

Medical professionals will first use the AI-generated recommendations as a preliminary reference. Then, they will search academic databases for relevant research literature or consult professional textbooks to cross-validate the AI results against these sources, thereby verifying the academic validity of the AI recommendations.

I’ll look up relevant literature on CNKI and compare the AI’s results with the content in the literature to see how accurate the AI is.

A2

If there’s a discrepancy with the textbook, I’ll go by the textbook—after all, it’s the authoritative source.

A4

#### Subtheme 2: Workflow Division Model

From health care professionals’ perspectives, optimizing workflow division is the foundation of efficient human-AI collaboration. AI systems handle standardized, repetitive preliminary tasks, while clinicians focus on personalized, complex clinical decision-making. This division reduces repetitive workloads, allowing clinicians to prioritize individualized care planning. Leveraging the complementary strengths of humans and AI improves time efficiency and decision quality and fosters win-win collaboration. Importantly, clinicians must critically assess AI hallucinations, verify AI-generated information, and exercise critical thinking when adopting AI-assisted recommendations.

Artificial intelligence helps me quickly process large volumes of basic data and provide preliminary diagnostic guidance. However, as healthcare professionals, we still need to conduct detailed patient interviews and physical examinations. AI recommendations can only serve as references.

A11

#### Subtheme 3: Professional Expertise-Driven Decision-Making

The principle of professional expertise-driven decision-making is the cornerstone of human-machine collaborative decision-making in the health care field, and it serves as the fundamental safeguard for ensuring that medical decisions align with clinical practice requirements and patient interests. In the practical application of AI-assisted decision-making, health care professionals always rely on their professional expertise as the ultimate criterion for decision-making.

AI only assists me in organizing patient information and generating preliminary reports. After completing the physical examination, I must make the final decision based on my own clinical experience and the latest research findings.

A14

### Theme 3: AI User Experience

#### Subtheme 1: Core Functional Scenarios

In daily clinical practice, AI tools have become important aids for clinicians to enhance work efficiency. We discovered that AI tools primarily handle repetitive tasks, effectively lessening clinicians’ routine workload and enabling them to concentrate more on core work that demands professional judgment. During diagnosis, AI tools use large-scale clinical data and medical knowledge to offer clinicians preliminary diagnostic suggestions and risk assessments, supporting a more comprehensive analysis of patients’ conditions and reducing the incidence of misdiagnosis and missed diagnoses.

Using AI tools to analyze imaging can identify and mark injury sites, indicate potential complications, and gain valuable time for subsequent resuscitation and treatment.

A22

Beyond clinical practice, AI tools also play a role in medical education and academic research. They support the development of documents and course materials, including outline generation and formatting, which improves the efficiency and quality of educational materials and academic literature. However, the use of AI in these areas must comply with relevant regulations and guidelines.

When preparing presentations, I use AI to select appropriate topics. Once the topic is set, AI helps design course frameworks and develop teaching ideas. This saves substantial preparation time and allows for further content refinement.

A5

#### Subtheme 2: Support Requirements

The large volumes of clinical data generated in daily practice require systematic organization and analysis. However, traditional methods are often time-consuming and labor-intensive. Health care professionals expect AI tools to integrate, analyze, and visualize these data.

It would be ideal if AI could automatically generate nursing notes; I would not need to write them one by one, only review and edit.

A1

I hope AI can achieve greater accuracy and depth in predicting disease progression.

A13

Effective deployment of AI tools relies on professional technical support. Clinicians expect expert guidance during use, timely problem resolution, and customized tool configuration to meet specific needs. Professional technical support not only assists clinicians with AI implementation but also clarifies AI regulations during training, helping to standardize clinical AI use and improve efficiency.

A dedicated technical support team should be established to ensure prompt resolution of technical issues.

A16

### Theme 4: Barriers to AI Adoption and Risk Concerns

#### Overview

Although AI has demonstrated significant value as an auxiliary tool, its widespread adoption still faces numerous barriers and risk concerns.

#### Subtheme 1: Data Risk Management

As the core foundation of AI tools, data quality and security directly determine the effectiveness and reliability of AI applications. Data risks mainly pertain to 3 aspects: completeness, accuracy, and security. In some AI tools, limited sample sizes fail to cover rare or special cases, reducing analytical accuracy and impeding effective support in complex scenarios. These are key barriers to broader clinical adoption.

If AI data sources are authoritative and updated in a timely manner, my trust will be further strengthened.

A14

Additionally, the sensitivity and criticality of health data require robust security mechanisms for AI tools. Clinicians expect AI to ensure data security, protect privacy, and enhance system stability. Safeguarding AI applications is fundamental to lawful use, and clinicians are responsible for protecting patient privacy and data security.

In my view, AI can only be used in healthcare when data security is guaranteed; we must ensure patients’ privacy is not compromised.

A10

#### Subtheme 2: Technical Capability Barriers

Technical barriers are another key factor limiting effective AI adoption. Current AI technologies have notable shortcomings, including a lack of standardized protocols, poor interoperability, and insufficient explainability. The absence of unified standards across AI tools increases usability challenges, while data exchange barriers between systems restrict AI’s full potential.

Hospitals should coordinate stakeholders to resolve system issues, ensure interoperability between AI information systems, and enable seamless workflow integration.

A6

Clinicians’ AI literacy directly impacts implementation outcomes. Interviews show that most staff want to improve their proficiency in using AI tools. Structured training is critical for building AI capabilities, but such resources are currently severely limited.

The support we need most is training. I think this is a very important issue.

A8

#### Subtheme 3: External Constraint Risks

External constraints primarily refer to institutional, regulatory, and oversight limitations that shape the use of AI. These factors are crucial for the safe and sustainable development of AI. Effective oversight mechanisms are essential safeguards for clinical AI, and robust regulatory frameworks are urgently needed.

Clear definition of liability for AI use is essential, especially to protect clinicians.

A10

Quality control of AI outputs is vital for clinical safety. Currently, effective quality assurance systems are lacking, which compromises AI reliability.

We need a comprehensive evaluation framework to regularly update and select high-quality AI tools.

A14

### Theme 5: Attitudes Toward AI Substitution and Perceptions of Core Health Care Values

#### Subtheme 1: The Irreplaceability of Health Care Professionals

The irreplaceability of health care professionals lies in 2 core dimensions: emotional and humanistic care, and clinical experience and comprehensive decision-making. Humanistic care is the core value of medical service and the human quality most difficult for AI to simulate. Health care professionals provide emotional support through nonverbal communication and verbal reassurance, and flexibly adjust communication styles for patients with diverse backgrounds and psychological states.

Despite rapid AI advancement, it cannot replace humanistic care, nor possess clinicians’ experience and judgment.

A14

The complexity and uncertainty of health care demand clinical decisions that are grounded in extensive practical experience and comprehensive judgment. Experienced clinicians make intuitive judgments in dynamic and complex situations. Their ability to anticipate risks is crucial in emergency resuscitation and critical care environments.

Even with another decade of development, AI will not anticipate unmanifested risks or deliver timely, precise resuscitation interventions.

A10

#### Subtheme 2: Consensus on Complementary Roles

Although health care professionals emphasize their irreplaceability, they do not reject AI technology. Instead, there is a consensus on complementary roles: AI provides auxiliary support, while professionals take the lead. This consensus extends beyond theory, driving the development of mature human-AI collaboration models in clinical practice. Participants in this study view AI as an assistive tool and clearly recognize its limitations. While AI can offer diagnostic suggestions, clinicians remain ultimately accountable for medical decisions.

The ideal scenario is for AI to handle repetitive, routine tasks, allowing healthcare professionals to focus on more specialized, innovative, and complex work.

A23

### Integrated Theoretical Model of AI Application Barriers

**Figure 2:**
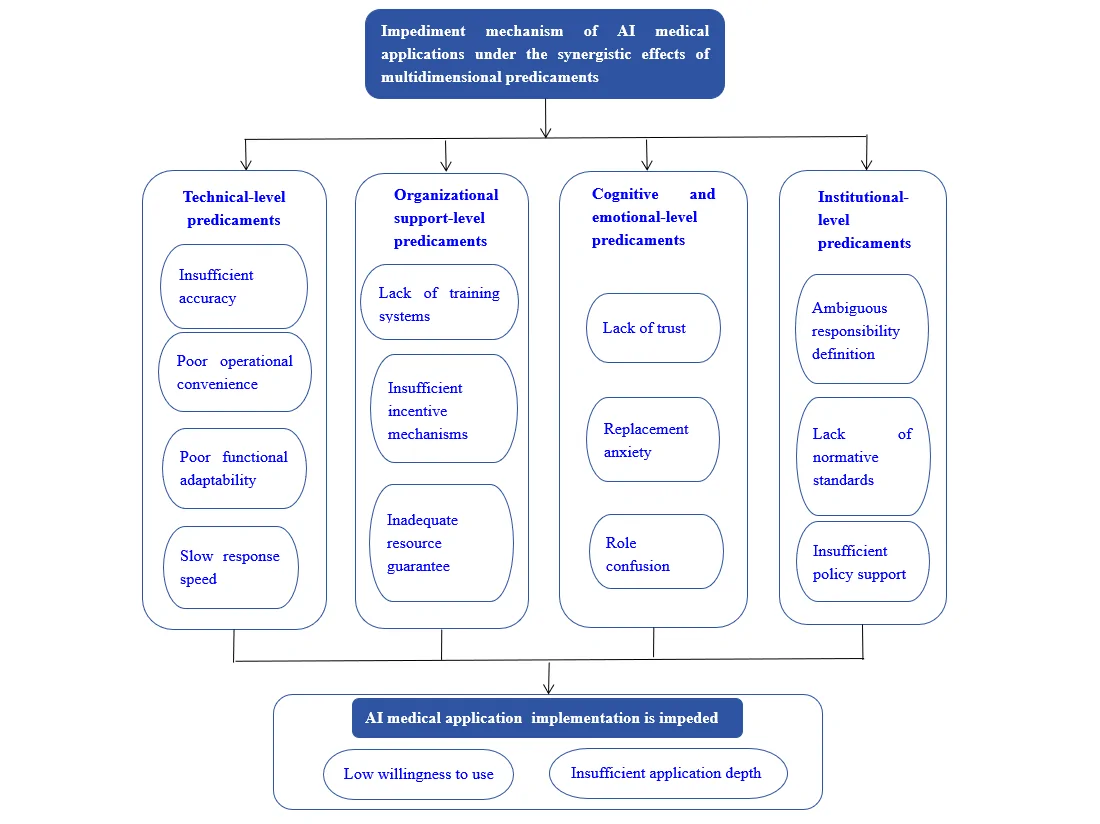
Theoretical model illustrating the formation mechanism of predicaments in AI applications.

Our results indicated that the obstacles to the implementation of AI in the health care sector stem from the synergistic interaction of multidimensional challenges. Based on this, a theoretical model of the challenges facing AI applications in health care was constructed (Figure 2). At its core is the mechanism of barriers to AI health care applications arising from the synergistic interaction of these multidimensional challenges, which is consistent with the conclusions of existing research that AI health care applications must overcome multilevel obstacles [15,16]. The challenges facing AI health care applications primarily encompass 4 major dimensions. These dimensions do not exist in isolation but rather form a dynamic mechanism characterized by interweaving, transmission, and mutual reinforcement.

## Discussion

### Principal Findings

Through semistructured, in-depth interviews, the study explored the multifaceted challenges and underlying mechanisms faced by 23 health care professionals, representing diverse roles, years of experience, and professional titles, during the implementation of AI in health care. It identified the dimensional characteristics of these challenges and group differences, providing empirical evidence to optimize the practical implementation of AI in health care. All research objectives were fully achieved. Through inductive thematic analysis, 5 themes and 13 subthemes were identified, revealing that the challenges of AI medical applications can be categorized into 4 dimensions: technology, organizational support, cognition and emotion, and institutional factors. Challenges across these dimensions intertwine to form a synergistic barrier mechanism, and significant heterogeneity exists in the challenges faced by different groups.

### Multidimensional Challenges of Health Care AI

Technical challenges pose a fundamental barrier to the adoption of AI in health care, primarily manifested in the current AI’s insufficient accuracy, ease of use, and functional adaptability. This finding is basically consistent with existing research [17,18]. The health care industry demands extremely high levels of technical precision. The limitations of AI in diagnosing complex cases and addressing personalized care needs have, to some extent, eroded clinicians’ trust and adoption intention. It may also exacerbate confusion over role positioning. Additionally, AI interface designs that do not align with operational habits and involve high learning costs further reduce usability.

Challenges at the organizational support level stem from a lack of safeguards for AI applications in health care. In addition to the training gaps highlighted in existing research [19], the study further found that insufficient incentive mechanisms and inadequate resource allocation are equally critical. Health care professionals are overburdened with work and lack additional motivation to use AI tools [20]. Insufficient organizational support makes it difficult for them to master these methods, resulting in low adoption rates [21]. At the same time, some hospitals lack the infrastructure to ensure the stable operation of AI tools, and technical maintenance is often delayed [22], further limiting the depth and breadth of AI applications.

Cognitive and emotional challenges constitute the primary subjective barriers to AI applications in health care, mainly manifesting as low trust, replacement anxiety, and role confusion. Health care professionals have difficulty understanding AI decision-making processes and question the reliability of the results [23]. Replacement anxiety stems from a job security crisis triggered by AI, especially among senior health care professionals who fear that their experiential advantages will be superseded [24]. Role confusion arises from unclear boundaries in human-machine collaboration [25] and a lack of clear standards for the division of labor. This is largely consistent with the study’s findings, further validating the direct impact of cognitive and emotional factors on AI adoption behavior.

Institutional challenges stem from regulatory gaps in AI health care applications, which are primarily characterized by ambiguous liability definitions, inadequate operational protocols, and data security standards. Since AI outputs and health care professionals’ decisions jointly influence clinical outcomes, it is difficult to allocate responsibility among parties in the event of a medical dispute [26]. Meanwhile, the absence of unified regulatory standards may deter health care professionals from using AI due to risk concerns.

### Considerations of Overreliance and Cognitive Risks

While participants in this study acknowledged the supportive value of AI, its potential long-term adverse effects must not be overlooked. A major concern is cognitive relinquishment: excessive reliance on AI outputs may lead to the atrophy of clinicians’ clinical reasoning, differential diagnosis, and critical thinking skills [25]. The principles of professional oversight and cautious use proposed by participants implicitly serve as intuitive safeguards against this risk.

Nevertheless, these principles may be compromised in fast-paced clinical settings, giving rise to decision-making inertia. In the long run, clinicians may experience a gradual deterioration of their professional competence. They could become proficient at operating AI systems but lose the habit of independent clinical thinking [26,27]. When AI malfunctions, provides recommendations for rare cases, or encounters patients with complex comorbidities, clinicians may lack adequate capacity for autonomous decision-making.

Furthermore, clinicians may adopt AI suggestions without fully understanding its underlying logic, which represents a form of cognitive outsourcing. This can erode professional expertise, authority, and self-confidence. Accordingly, the design of clinical AI tools and relevant training programs in the future should aim to augment rather than replace human intelligence, instead of merely pursuing higher efficiency. Medical education needs to strengthen training in critical thinking amid AI adoption and incorporate AI interpretation and rigorous questioning into core clinical competencies. Clinicians should remain the ultimate decision-makers and retain their core professional capabilities at all times.

### Definitions of Key Concepts

The key concepts addressed in the findings of this study, AI literacy, interoperability, and safety, are further elaborated below.

AI literacy [28] refers to the comprehensive ability of health care professionals to correctly understand, proficiently operate, and effectively apply AI tools, as well as to effectively identify and mitigate associated risks. It encompasses an understanding of underlying principles, operational skills, critical thinking, and risk awareness. In this study, insufficient AI literacy represents a significant challenge at the organizational level, particularly among junior-level professionals. Their lack of systematic training results in more pronounced deficiencies in AI literacy.

Interoperability [29] refers to the ability of AI tools and devices, as well as different health care information systems and applications, to share data across organizations and scenarios. Our findings indicate that poor compatibility between AI tools and existing professional equipment and technical standards, along with inadequate data sharing, not only undermines the performance of AI applications but also increases the workload of management staff.

Security [30] primarily refers to the stability, accuracy, interpretability, lack of bias, tamper resistance, and privacy protection of AI tools in clinical applications, ensuring they do not lead to risks such as misdiagnosis, missed diagnosis, data breaches, or liability disputes. The study results indicate that current ambiguities in liability determination and incomplete data security standards expose health care professionals to potential risks, thereby hindering the widespread adoption of AI.

### Group Differences in AI Challenges

This study found that the challenges faced in AI application vary significantly across different groups due to differences in job roles, work experience, and professional qualifications. This provides a crucial basis for effectively overcoming barriers to AI adoption.

Regarding job role differences, the nature of work in different positions determines the specificity of AI application needs and challenges, which is largely consistent with existing research findings that job role differences influence the AI application experience [31]. Clinicians are more concerned with AI accuracy and the delineation of responsibility [32], worrying about the risk of misdiagnosis; nurses face issues related to the cumbersome operation of AI-enabled nursing devices and their disconnect from established nursing workflows; medical technical staff focus on the compatibility of AI with existing equipment and the professionalism of the results [33], while management and administrative staff face the dual challenges of data sharing and privacy protection [34].

Regarding differences in years of experience, young practitioners (≤5 years) exhibit strong learning abilities and high receptiveness [35], yet they lack AI operational skills and application experience. Middle-aged practitioners (6-15 years), as the backbone of the workforce, find it difficult to adapt to AI integration because of their established work patterns [36]. Moreover, their declining learning ability further intensifies the adaptation pressure. Senior practitioners (≥16 years) have high professional authority but low receptiveness. They believe their own experience surpasses that of AI and even hold biases. This pattern aligns with the career development trajectory of health care professionals.

Regarding professional title differences, individuals with junior titles find it difficult to access sufficient training resources [37] and have a weak foundation in AI application. Those with intermediate titles encounter a mismatch between their needs and AI capabilities [38] and lack the motivation to adopt the technology. Individuals with associate senior titles and above demonstrate low trust in AI, believing that it has a limited understanding of complex clinical scenarios [39], and they refuse to rely on AI for core work processes. This gradient reflects the intrinsic link between professional rank, the demand for AI application, and the level of trust in it.

### Integration of Challenge Causes and Targeted Solutions

Based on the multidimensional challenges and cross-group differences discussed earlier, we further integrate the underlying causes of these challenges and propose targeted solutions: The challenges across the 4 dimensions do not exist in isolation but are intertwined, interconnected, and mutually reinforcing, forming a dynamic mechanism of obstruction. At the technical level, issues such as insufficient accuracy and data security risks erode practitioners’ trust [3]. This phenomenon is particularly pronounced among those with fewer years of experience and lower professional titles [40]. At the organizational level, resource shortages constrain the implementation and iterative application of technology [41], leading to a vicious cycle where technological adoption is further hindered. At the institutional level, the absence of standards exacerbates technological risks, intensifying practitioners’ safety concerns and solidifying cognitive barriers.

To address these challenges, we must ground our approach in both common root causes and group-specific differences while adhering to targeted measures. At the job level, AI products must be custom-developed, with clear delineation of responsibilities among stakeholders [42] and optimized human-machine collaboration processes. A cross-departmental collaboration and application effectiveness evaluation system should be established to specifically address core issues such as insufficient technological adaptability and lack of organizational support. At the group level, AI training and practical support should be strengthened for junior staff and those with entry-level professional titles to address gaps in AI literacy. For mid-career professionals, we should guide them to integrate their experience, alleviate anxiety about job displacement, and boost their enthusiasm for AI adoption. For senior professionals, we should encourage them to update their knowledge, play a leading role in the industry, and eliminate biases against AI technology.

Based on insights from practical development, medical institutions must focus on these challenges and demographic differences. They should introduce and develop AI products tailored to specific needs, improve organizational safeguards and tiered training systems, strengthen compliance management and data security protection [32], and ensure open channels for feedback from practitioners. Policymakers must refine regulations and standards for AI in health care and implement end-to-end oversight [43]. They should establish collaborative platforms involving health care institutions, research organizations, and AI companies, and promote technological research and development, the commercialization of research outcomes, and talent development to ensure the effective implementation of AI in health care.

### Limitations

This study has the following limitations. First, the sample size is limited. The data sources are relatively concentrated, and the transferability of the findings is limited. The findings may not be directly applicable to other clinical settings or populations with different AI implementation contexts. Second, the trusting relationship established during interviews may have led to response bias. Therefore, a neutral stance was maintained throughout the interviews, leading questions were avoided, and reflective field notes were recorded after each interview. Third, the study focuses on the subjective experiences of health care professionals and does not incorporate objective data on the effectiveness of AI medical applications into the analysis. Fourth, potential subjective biases during the coding process cannot be completely ruled out. To minimize bias, this study employed independent dual coding, reflective notes, and group discussions to reach consensus and established a complete audit trail. Lastly, the study did not explore the impact of factors such as AI technology types and hospital levels on the challenges of application.

### Conclusions

Through systematic investigation, this study has identified that challenges associated with AI adoption in health care are multidimensional and interrelated, with notable variations across different groups. These challenges can be categorized into 4 interconnected dimensions that collectively form an obstructive mechanism. The difficulties encountered by health care professionals differ according to their roles, work experience, and professional titles. Accordingly, tailored solutions are recommended rather than one-size-fits-all strategies. While pursuing efficiency gains from technological empowerment, potential cognitive risks and value distortion must be guarded against. Participants’ rational perceptions of AI and demands for professional oversight essentially reflect proactive precautions against overreliance and the gradual erosion of clinical competencies. Hence, future practice and research should not merely focus on removing barriers to AI implementation, but also foster a development paradigm that augments rather than replaces human intelligence. The findings further enrich theoretical research on barriers to AI health care applications, refine the theoretical framework of multidimensional challenge synergy mechanisms, and provide practical guidance for optimizing the implementation of AI in health care. This study holds significant implications for research, clinical practice, education, management, and policy.

Theoretically, the collaborative framework established in this study lays a foundation for future research and facilitates further exploratory studies on the relative weight of various influencing challenges. In clinical practice, it provides a basis for optimizing AI medical application workflows, enabling the development of customized AI products and the clarification of boundaries and responsibilities in human-machine collaboration. In education, a training system can be developed to address gaps in AI literacy, targeting young professionals and entry-level individuals. In management, organizational safeguards, evaluation, and feedback systems within medical institutions can be improved. In policy, institutional regulations and oversight can be strengthened to promote multistakeholder collaboration. To address the limitations of this study, future research may expand the sample size, track the evolution of AI applications in health care, and analyze emerging challenges and corresponding solutions, so as to advance the high-quality development of medical AI.

## Appendix

Multimedia Appendix 1 Semistructured interview guide.

## Abbreviations

COREQ: Consolidated Criteria for Reporting Qualitative Research

## Data Availability

The data generated and analyzed during this study are not publicly available due to privacy and ethical restrictions related to participant confidentiality. Anonymized processed data are available from the corresponding author upon reasonable request.
