# Supplementary material for: The Challenges of AI Applications in Health Care: Qualitative Study
Source: J Med Internet Res. 2026 Aug 18;28:e92979. doi: 10.2196/92979 (PMC13535307; doi:10.2196/92979)
Supplement: Supplementary file 1 [file jmir_v28i1e92979_app1.docx]

**Appendix 1: Semi-Structured Interview Guide**

**I. General Information**

1. Demographic Information: Age, Gender, Highest Level of Education

2. Professional Background Information: Job Category, Professional Title Level, Years of Experience, Department Affiliation

3. AI Usage Experience: Types of AI Tools Used, Frequency of Use, Date of First AI Exposure

**II. Current AI Usage: User Experience and Evaluation**

1. Core Experience: Describe a specific scenario where you most frequently use AI tools in your daily work. What key problems does it help you solve?

2. Technical Evaluation: During your usage, how would you rate the tool's accuracy, response speed, and ease of operation? Can you share a specific positive or negative example?

3.Decision Conflict: When AI outputs conflict with your professional judgment, how do you proceed? How does this impact your decision-making?

**III. Perceived Challenges: Key Difficulties and Obstacles**

1. Core Barriers: What do you consider the biggest obstacle currently preventing you from using AI tools more effectively? (Is it the technology itself, insufficient training, process issues, or other reasons?)

2. Trust and Concerns: How much do you trust AI tools? What factors increase or decrease your trust? Are you concerned that AI might replace your job?

**IV. Optimizing Needs and Expectations**

1. Feature Enhancements: If possible, in which areas would you most like to see improvements in AI tools? (e.g., greater usability, increased transparency, enhanced accuracy)

2.Support Requirements: What support do you believe hospitals should provide (e.g., training, incentive policies, clear delineation of responsibilities) to make you more willing and confident in using AI?

**V. Future Outlook — Future Vision and Role Evolution**

1. Vision for Human-Machine Collaboration: In your ideal scenario, how should AI and healthcare professionals divide tasks and collaborate? Describe a “human-machine synergy” work scenario you envision.

2. Irreplaceable Value: What core values in medical practice do you believe AI will never be able to replace in humans? Why?
